# SIgA in various pulmonary diseases

**DOI:** 10.1186/s40001-023-01282-5

**Published:** 2023-08-27

**Authors:** Xintian Wang, Jun Zhang, Yan Wu, Yuncong Xu, Jinxu Zheng

**Affiliations:** 1 Department of Respiratory Medicine, Affiliated Hospital of Jiangsu University, No. 438, Jiefang Road, Jingkou District, Zhenjiang, Jiangsu China; 2 Department of Respiratory and Critical Care Medicine, Aoyang Hospital Affiliated to Jiangsu University, No. 279, Jingang Avenue, Zhangjiagang, Suzhou, Jiangsu China

**Keywords:** Secretory immunoglobulin A, Lung disease, Respiratory, Mucosal immunity

## Abstract

Secretory immunoglobulin A (SIgA) is one of the most abundant immunoglobulin subtypes among mucosa, which plays an indispensable role in the first-line protection against invading pathogens and antigens. Therefore, the role of respiratory SIgA in respiratory mucosal immune diseases has attracted more and more attention. Although the role of SIgA in intestinal mucosal immunity has been widely studied, the cell types responsible for SIgA and the interactions between cells are still unclear. Here, we conducted a wide search of relevant studies and sorted out the relationship between SIgA and some pulmonary diseases (COPD, asthma, tuberculosis, idiopathic pulmonary fibrosis, COVID-19, lung cancer), which found SIgA is involved in the pathogenesis and progression of various lung diseases, intending to provide new ideas for the prevention, diagnosis, and treatment of related lung diseases.

## Introduction

SIgA is a kind of immunoglobulin A (IgA) antibody, which is mainly distributed in milk, tears, saliva, airway, gastrointestinal secretions, and other mucosal secretions, playing an important role in mucosal immune response, tumor formation, anti-allergy, and other aspects. For the respiratory system, with every breath, we inhale thousands of particles into the airway, posing many potential threats to the integrity of the lungs. The airway mucosal immune system serves as a powerful protective mechanism, providing innate and adaptive responses to these inhaled particles, inflammatory responses to harmful antigens, and tolerance mechanisms to harmless antigens. The failure of these responses may lead to increased antigen infiltration and repeated infections, or excessive immune response to harmless antigens, all of which may lead to chronic airway disease and a series of related lung diseases [1]. In this review, we summarized relevant information on SIgA mucosal immune system and investigated how its dysfunction affects the pathogenesis and clinical course of a series of lung diseases.

## Overview of SIgA

### Synthesis of SIgA

Typical SIgA molecules are composed of IgA, J chains, and secretory components (SC). IgA and J chains are synthesized from plasma cells, which are located in the lamina propria of the respiratory tract, gastrointestinal tract, urogenital tract, and other mucous membranes. After synthesis, one J chain connects two monomer IgA to form dimer IgA (dIgA). After the formation of dIgA, it is secreted from plasma cells and forms a dIgA–pIgR complex with the polymeric immunoglobulin receptor (pIgR) expressed by epithelial cells [2]. Subsequently, under the action of proteolytic enzymes, the extracellular segment of pIgR, namely, SC, combines with dIgA and finally forms SIgA [3, 4].

### Function of SIgA

SIgA is the main antibody involved in local immunity. As the main responding factor of the mucosal immune system, it plays an indispensable role in resisting the invasion of external pathogenic microorganisms. It not only neutralizes bacterial toxins but also participates in the formation of the immune barrier and immune clearance. It can affect the progress of autoimmune diseases by mediating immune response [5].

### Factors affecting the level of SIgA

Studies have shown that SIgA level may be related to the level of steroid hormones. In particular, there is a remarkable positive correlation between the sex hormones, such as testosterone and estrogen and SIgA [6]. The level of SIgA is also regulated by different types of immune cells. T lymphocytes participate in the production of IgA. B lymphocytes, micro pleated epithelial cells (M cells), and the molecules expressed by M cells (activating inducible cytidine deaminase and TGF‐β) are also essential for the production of IgA [7, 8]. In addition, various cytokines and the external environment can also regulate the level of SIgA. Studies have shown that there is a relationship between SIgA production and cytokine production [9], TNF-β can up-regulate the expression of fpIgR and the production of SC, which is modulated by PI3K and NF-κB signal pathway [10]. IL-10, IL-4, retinoic acid, and IgA-induced proteins were also identified as stimulators of IgA production [11]. In addition, it has been found in recent years that cold exposure may inhibit the production of B lymphocyte activating factor, leading to the inhibition of IgA secretion in bronchial epithelium, thus increasing the frequency of acute viral respiratory infection in cold weather. Therefore, it is suggested that temperature can affect mucosal immune function by regulating the production of SIgA [12].

## The relationship between SIgA and pulmonary diseases

**Fig. 1:**
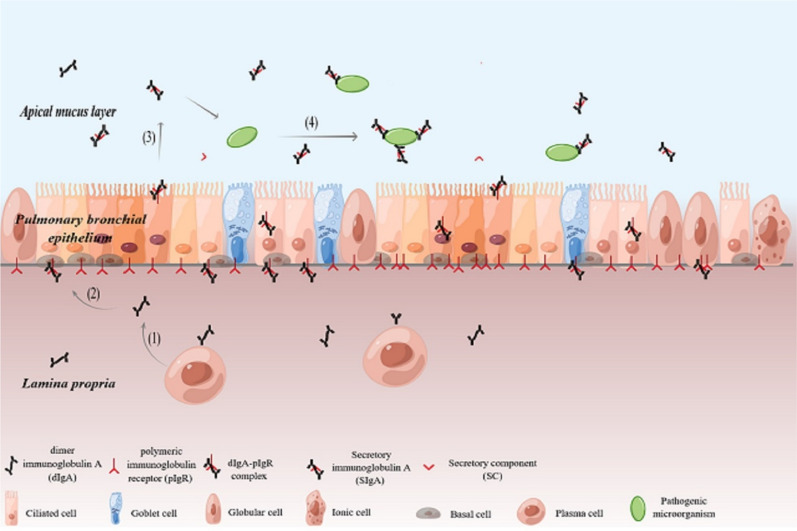
Production of SIgA in lung epithelial mucosa and its role in resisting the invasion of external pathogenic microorganisms. (1) Plasma cells in the lamina propria secrete dIgA. (2) DIgA binds to pIgR produced by pulmonary bronchial epithelial cells and stimulates endocytosis to form dIgA pIgR complex. (3) Under the action of proteolytic enzyme, the extracellular segment of pIgR, namely, SC, combines with dIgA to form SIgA, which is released into the top mucus layer. (4) SIgA drives immune rejection, agglutinates, intercepts and eliminates pathogenic microorganisms*.* Acknowledgements: the figure is drawn by Figdraw

Pulmonary bronchial epithelium is a pseudostratified epithelium composed of many cell types, and its proportion is strictly controlled [13, 14]. Bronchial airway epithelium is mostly composed of ciliary and goblet cells. In addition, it also includes basal cells, globular cells, neuroendocrine cells, and rare ionic cells [15]. Ciliary and goblet cells work together to remove foreign particles and other irritant stimuli of the air spaces by Mucociliary transport or clearance. Among them, Goblet cells account for 5–15% of the airway epithelial cells of healthy people and their main function is to produce mucus. Goblet cells, are mostly absent in small airways. Ciliary cells are the most prominent of all epithelial cells, accounting for over 50% of airway epithelial cells. The synchronous swinging of their cilia produces the mucus layer to the trachea and throat [16]; Basal cells are pluripotent stem cells, whose proportion in respiratory bronchioles is relatively less compared to trachea [17], They not only strengthen the epithelium and the underlying lamina propria, but also help promote the steady and regular regeneration of epithelium after injury [18, 19]; Globular cells are involved in host defense, accounting for 20% of small airway epithelial cells [20, 21]; Neuroendocrine cells are rare [22], which are considered to be related to oxygen induction, smooth muscle tension and immune response [23, 24]; The rare ionic cells seem to control the viscosity of fluid and mucus on the airway surface [25]. Among them ciliary cells, goblet cells, globular cells and ionic cells have been shown to express pIgR, take part in the process of d-IgA formation and promote SIgA secretion to the apical mucus layer [26–28] (Fig. 1). Along the airway epithelium, SIgA helps to provide first-line mucosal protection against inhaled particles and pathogens. The dIgA produced by mucosal Plasma cell is transported to the apex of airway epithelial cells through pIgR, where it is released as SIgA. SIgA mediated immune response is involved in a series of lung diseases [1]. In Table 1, we summarized the association between SIgA mediated mucosal immune changes and SIgA dysfunction with the most common lung diseases (Table 1).

**Table 1:** Overview of SIgA levels in pulmonary diseases and the significance of impaired SIgA response

Disease	SIgA-levels	Cause of altered SIgA levels	Implication	References
COPD	Reduced	Downregulation of pIgR mediated by TGF-β	Higher risk of disease deterioration	[1, 29–31]
Asthma	Reduced	Downregulation of pIgR mediated by IL-4 and IL-2	Reduced immune rejection of allergens and immune regulatory effects on dendritic cells	[1, 32–35]
Pulmonary tuberculosis	Reduced	Impaired immune response to *Mycobacterium tuberculosis*	Impaired function in inhibiting pathogen adhesion and clearing pathogens, weakened protective effect of antibodies	[36–39]
IPF	Elevated	Ectopic lymphogenesis composed of a large number of IgA B lymphocytes	Promoting the proliferation and collagen contraction of human lung fibroblasts	[40–43]
COVID-19	Severe course when reduced early and increased in later stages of diseases	Impaired immune response to SARS-CoV-2	Mucosal virus clearance impaired, neutrophil inflammatory activation enhanced	[44–49]
Lung cancer	Increased in normal immune system and decreased in deficient immune system	Body autoimmunity	Mucosal immune surveillance was normal or weakened	[50, 51]

### SIgA and chronic obstructive pulmonary disease

Chronic obstructive pulmonary disease (COPD) is a chronic and complex respiratory disease characterized by persistent airflow limitation and respiratory symptoms [52]. Modern medical research finds that decrease or lack of SIgA secretion in respiratory epithelium plays a role in the pathogenesis of COPD, due to a dysfunction of local immune response [29, 30, 53]. The destruction of the SIgA immune barrier will cause a pathological crosstalk cycle between the immune system and its adaptive branches, making COPD patients unable to normally drive the activation of adaptive immunity [54]. The loss of the SIgA immune barrier in small airways of patients with severe COPD is a complex process, which may be caused by pIgR-dependent defects in IgA transfection and SIgA degradation [27]. The lack of pIgR/SIgA in the airway will result in the continuous activation of the innate immune response, leading to progressive small airway remodeling and emphysema [55]. Gohy et al. linked the down-regulation of pIgR with bronchial epithelial reprogramming driven by TGF-β [31]. The inflammatory environment of COPD patients’ lungs is initiated by chronic infiltration of neutrophils. Inflammation leads to downregulation of pIgR through TGF, causing impaired IgA transport and subsequently a decrease in SIgA levels on the mucosa, and eventually contributing to the impaired lung IgA immune function in COPD patients [1, 56]. Polosukhin et al. found that the expression of pIgR in the bronchial epithelium was reduced, and the IgA secreted in bronchoalveolar lavage was also reduced in the bronchial mucosa area covered by the pseudostratified epithelium of COPD patients [53]. This may be connected with the reduced expression of pIgR gene in ciliated cells in the airway [57]. In COPD, the down-regulation of pIgR is clearer in the remodeling area of bronchial epithelium [53]. Richmond et al. found that pIgR-deficient mice lacking SIgA would spontaneously develop into mice with COPD-like changes in their bronchial epithelium with age [55].

In addition, the reduction of SIgA or pIgR levels is also related to the severity of COPD. A study found, the level of pIgR in the bronchial epithelium of severe COPD patients was lower than that in the control group, and the pIgR and IgA transport in the bronchial epithelial cell of severe COPD patients was reduced [31], This may be related to the chronic accumulation of CD8+ T lymphocytes in the small airways. Compared with the IgA positive airways, the intraepithelial and submucosal CD8+ T lymphocyte infiltration in the airways with IgA deficiency increases [53]. To our knowledge, there is no direct study on whether regional bronchial IgA scarcity is related to the rate and intensity of COPD exacerbations. However, an observational SPIROMICS cohort study showed that COPD patients with significantly reduced IgA levels of serum experienced more frequent and severe deterioration [29]. Since the lack of SIgA on the small airway surface is related to the progress of COPD, it can be considered a potential new therapeutic target for COPD.

### SIgA and asthma

Asthma is a chronic bronchitis disease characterized by chronic airway inflammation, airway remodeling, airway hyperresponsiveness, and elevated serum allergen-specific IgE concentrations. As an important factor involved in inflammation and regulating immunity, SIgA goes together with the occurrence and development of asthma. It may be used as an index to evaluate the susceptibility, severity, and treatment effect of asthma.

Some scholars found that the level of serum IgA was positively correlated with the results of asthma control test, and the IgA level of asthma patients has significant negative correlation with the prevalence of moderate–severe asthma. With the increase of serum IgA level, the severity of asthma could be better controlled and improved [58]. SIgA levels can be induced by different stimuli, such as cytokine IL-4 [59], or TGF produced by dendritic cells-β [60]. In asthma, pIgR expression is downregulated due to the release of IL-4 and IL-13 by Th2-cells [32]. The decrease in mucosal SIgA leads to a decrease in immune rejection of allergens, while the immune system’s allergic response is enhanced; In addition, the immune regulatory effect of SIgA on dendritic cells and subsequent inflammatory suppression are impaired [1, 33]. In a study done by Shkalim et al. among children in Israel, it was found that that asthma was the most common allergic disease in patients with selective immunoglobulin A deficiency (SIgAD), based on evaluation of their clinical and immunological characteristics [61]. This may be related to the fact that patients with SIgAD cannot neutralize or prevent the absorption of allergens so they are prone to allergic diseases, such as asthma and food allergy [34]. Urm et al. conducted a case–control study and found that asthma patients are more susceptible to SIgAD than non-asthma patients, and SIgAD increases the risk of bacterial infections and recurring infections among patients with asthma. It was also found that an elevated white blood cell count is associated with low levels of serum IgA in asthma patients with recurring infections [34, 61–64]. However, some studies have concluded that SIgAD, as an immunodeficiency disease, may be related to increased serum IgE levels [61]. Shahin et al. have also reached a similar conclusion. They have revealed serum IgA level is correlated with serum IgE level, and the correlation is significantly negative [65]. Several studies have confirmed that serum IgE levels of asthmatic patients are elevated [66, 67]. This further shows that the progression of asthma is closely related to SIgA.

In spite of all its protective effects on infectious, allergic and autoimmune disease, some studies have shown that SIgA has some negative effects on human biology. For instance, because of its function as esonophil and basophil activator, SIgA can perpetuate allergic reactions and exacerbations of asthma, although this is supposed to have a protective effect on parasitic infestations [68, 69]. Further research is needed to understand the correlation of SIgA levels to severity of asthma. Overall, can be a potential target for treatment of asthma in future.

### SIgA and tuberculosis

Pulmonary tuberculosis is a potentially fatal infectious disease caused by *Mycobacterium tuberculosis*. A growing number of studies have shown a protective effect of SIgA on various viral and bacterial infections, such as tuberculosis [70]. SIgA protects the mucosal epithelial barrier through different mechanisms [39]. On one hand, it can prevent bacteria from entering the lung by preventing bacterial antigens from adhering to the mucosal surface through the SC [37, 39, 71]. On the other hand, it can accelerate the clearance of immune complexes through respiratory ciliary movement [72], and stimulate antigen-presenting cells to activate T cells [38], thus activating protection against *Mycobacterium tuberculosis* infection [73]. However, When the body's immune system declines, the strong mucosal immunity cannot trigger a decrease in SIgA production, making the body more susceptible to infection by *Mycobacterium tuberculosis* [36].

In mice, infected with *Mycobacterium tuberculosis*, SIgA has shown to induce formation of great organized granuloma, which is a sign of effective immune activation and disease control. SIgA has also shown to greatly reduce bacterial load of lung and as a consequence the damage to lung is minimized [70, 74]. Respiratory immunization with a DNA vaccine prepared with chitosan can protect tuberculosis by enhancing the production of SIgA in lung bronchoalveolar lavage fluid (BALF) [75, 76]. NP, a potential new nanoparticle adjuvant of the tuberculosis subunit vaccine, can also increase SIgA and other immune proteins in lung lavage fluid to induce strong cellular and humoral immune responses [77]. Mice with reduced IgA and pIgR levels were more susceptible to BCG infection [78]. Rodriguez et al. showed that IgA-deficient mice immunized with Mycobacterium cell surface antigen were more susceptible to intranasal BCG infection than wild-type mice [78]. Further research is needed to understand if the stimulation of respiratory SIgA would modulate the pathogenic effects of *Mycobacterium tuberculosis*. This remains an important yet unexplored area.

### SIgA and idiopathic pulmonary fibrosis

Idiopathic pulmonary fibrosis (IPF) is the most common disease in idiopathic interstitial pneumonia. It has a poor prognosis and is a fatal respiratory disease [79], characterized by excessive deposition of fibrous tissue in the alveolar septal area [41, 80].

Some studies have shown that SIgA may promote the proliferation of human lung fibroblasts by promoting inflammation by induction of various cytokines, such as interleukin (IL)-6, IL-8, monocyte chemoattractant protein (MCP)-1 [81], thereby further promoting the proliferation of human pulmonary fibroblasts and collagen contraction [40]. The excessive proliferation of lung fibroblasts will in turn results in excess production of extracellular matrix and thereby promoting human lung fibrosis [40, 82]. In addition, Mota P et al. proposed that the bronchiolar epithelium secretes IgA into the airway cavity through pIgR, forming mucus rich in SIgA, which accumulates in the IPF lung [41, 42]. It is worth noting that the accumulation of this mucus is related to the decrease of FVC. Some studies have shown that SIgA can bind to the surface of A549 cells. A549 is usually used as a cell line with AT2 cell phenotype in vitro experiments, which can promote the production of vascular endothelial growth factor (VEGF), and transforming growth factor (TGF)‐β [83]. Notably, these cytokines are involved in the pathogenesis of IPF [84–86]. In addition, research suggests that TGF‐β is the strongest cytokine for fibrosis formation in IPF [87], and is crucial for the production of SIgA. In addition, it can induce conversion of B lymphocytes to IgA production. Therefore, an environment rich in TGF‐β may increase the number of IgA lymphocytes and the concentration of IgA. In the lungs of IPF patients, a tertiary lymphatic structure can be observed, which is the formation of ectopic lymphocytes composed of a large number of IgA B lymphocytes. This circulating feedback leads to an increase in sIgA levels in the lungs of IPF patients [43]. However, the origin of fibroblasts accumulated in IPF remains elusive. Epithelial mesenchymal transition (EMT) is a possible mechanism [88]. In EMT, airway epithelial cells and alveolar epithelial cells located near the bronchiole lumen acquire mesenchymal characteristics and become fibroblasts [89], and these cells react to SIgA in airway mucus after coming in contact with it, thus intensifying the formation of fibrosis [90]. Some research shows that this may be related to TGF-β/Smad pathway [91]. Therefore, this pathway can be used as a target for treating the pulmonary interstitial fibrosis. In the bleomycin-induced pulmonary fibrosis mouse model, dasatinib, an inhibitor of the TGF-β/Smad pathway, can inhibit the EMT of mouse alveolar and bronchial epithelial cells, thereby slowing down the progress of pulmonary fibrosis by reducing the reaction with SIgA [91]. In addition, Ten Klooster L et al. found, that serum IgA level can be treated as a prognostic biomarker of IPF, and high serum IgA level indicates a worse prognosis [92]. The above studies fully show that SIgA is significantly related to the progress and prognosis of IPF patients.

In addition, studies have shown that SIgA can promote the occurrence and development of IPF due to its ability to bind to transferrin receptor (TfR) CD71, thereby blocking the binding of SIgA and CD71 [40]. This also provides a new target for the treatment of IPF. However, other studies show that CD71 does not seem to be the only receptor involved. Because as a key receptor for human life, CD71 is necessary for both cell iron introduction and cell maintenance. Therefore, it seems that it is not feasible to eliminate the effect of SIgA on some fibrogenic diseases by targeting CD71, and its feasibility needs to be further studied [93]. Recently, it has been found that ANXA2, which is expressed in airway epithelial cells, is a new receptor for SIgA. Immunohistochemistry of lung sections shows that ANXA2 is clearly expressed in airway epithelial cells [94]. Further research is needed to explore the interaction between ANXA2 or other possible receptors and SIgA in IPF, which can provide new ideas for our treatment of IPF.

### SIgA and COVID-19

The COVID-19 pandemic caused by severe acute respiratory syndrome coronavirus 2 (SARS-CoV-2) has carried a large threat to public health. The main transmission route of SARS-CoV-2 is inhaling respiratory droplets which contain virus particles [95], inducing the body to produce SIgA antibodies in saliva, nasal juice, and nasal cavity, thereby activating mucosal immune response and limiting the virus to the upper respiratory tract, resulting in asymptomatic infection or only mild symptoms [49]. SIgA plays a part in antiviral protective immunity. The determination of IgA in saliva and serum as a marker deserves further study in SARS-CoV-2 infection [96].

It has been found that SIgA in the saliva of severe COVID-19 patients is also reactive to non-novel coronavirus, and this heterologous immune response consists of a non-protective cross-reaction [97]. Similarly, another study showed that cross reactive SARS-CoV-2 SIgA also existed in the saliva of people non-infected with the SARS-CoV-2 virus, indicating that SIgA may be helpful in preventing SARS-CoV-2 infection [98]. An investigation found that the positive rate of SARS-CoV-2 reactive saliva IgA antibody in Japanese people not infected with novel coronavirus was unexpectedly high, which may be an important reason for the low prevalence of COVID-19 in Japan [99].

There is evidence that the susceptibility to SARS-CoV-2 infection increases with age, the positive rate of reactive saliva IgA antibody in minors is higher than that in adults, which helps reduce their susceptibility to the virus [99]. In contrast, the susceptibility of the elderly is much higher [100, 101], which may be related to the decrease of SARS-CoV-2 reactive saliva IgA antibody with age [102], or the lack of mannose-binding lectin [103]. These studies indicate that individuals with a lack of saliva IgA antibodies, or individuals with negative reactions between saliva IgA antibodies and SARS-CoV-2, have a high risk of virus infection [104], which may also be the reason for the ineffectiveness of severe COVID-19 vaccine [105].

There are other routes of SARS-CoV-2 transmission. For example, the gastrointestinal tract may also be an important entrance or interaction site, and the role of the intestinal mucosal immune system as the first line of physical and immune defense is crucial [106]. In terms of vertical transmission, injecting the SARS-CoV-2 mRNA vaccine during pregnancy and lactation can induce an anti-SARS-CoV-2 IgA reaction in milk [107]. The SARS-CoV-2-infected mother is in good health and can start or continue breastfeeding [95]. Some studies even advocate that breastfeeding can be maintained during infection, because the SARS-CoV-2-specific antibody and free secretory component (fSC) secreted in breast milk can provide passive immunity for infants [108], and protect them from COVID-19 disease and gastrointestinal-related diseases [109, 110].

The most effective measure to control the COVID-19 pandemic is to vaccinate against SARS-CoV-2. The systemic and mucosal IgA responses caused by mRNA vaccination are different, so the effect of preventing subsequent infection is also different. The new SARS-CoV-2 is constantly changing, and the development of the COVID-19 vaccine that causes high levels of IgA may reduce human-to-human transmission [111]. In individuals not previously exposed to SARS-CoV-2, vaccination can induce minimal mucosal SIgA reaction, while in patients with a history of COVID-19, SIgA induction after vaccination is more effective [112]. The widespread use of vaccine preparations is likely to be very useful in determining the individual immune status of patients infected with SARS-CoV-2 or vaccinated [113]. Similar studies have found that vaccine adjuvants can also affect the mucosal immunity of the respiratory tract to COVID-19 by affecting the level of SIgA. Cao et al. believe that the recombinant virus encoding the trimeric SARS-CoV-2 spike receptor binding domain can produce a high level of serum anti-SARS-CoV-2 pseudovirus IgA and alveolar lavage fluid RBD-specific SIgA, causing a strong systemic immune response and mucosal neutralizing antibodies, which is expected to be used as a new SARS-CoV-2 vaccine [114]. Zheng et al. found that the inhalable nanovaccine with a bionic coronavirus structure can bind to the coronavirus by secreting high titer SIgA, thus triggering the mucosal immunity of respiratory tract to COVID-19 [115]. In addition, it has been suggested that SIgA in BALF of mice inoculated with live attenuated vaccine d16 developed with SARS-CoV-2 lacking nsp16 can effectively activate mucosal immunity against SARS-CoV-2 [116]. As a potential protective factor, Vitamin A have an effect on SARS-CoV-2 infection because of the fact that retinoic acid, the active metabolite of vitamin A, can mediate the production of SIgA in the respiratory tract, thus exerting the immune regulation function [117]. In addition, The supplement of neutrophil elastase inhibitor can promote the development of mucosal immunity including SIgA [118, 119], by stimulation of B cell activating factor, proliferation inducing ligand and IL-10 of TNF family [120], it can help to produce SIgA to prevent infection of pathogens at one or more mucosal sites, which is of great significance for the development of future vaccines.

In addition to the beneficial effects of mucosal IgA response in preventing SARS-CoV-2 infection, the harmful effects of IgA in COVID-19 have also been observed. Especially in the later stages of COVID-19, when the virus neutralizing activity of SIgA is lost, the result may be fatal [47, 121]. For example, Staats et al. described the correlation between anti SARS-CoV-2 IgA antibody in the serum of severe COVID-19 patients and the formation of Neutrophil extracellular traps (NET). They found that subclass IgA antibodies, in particular, are effective activators of neutrophil inflammation and NET formation [122]. In COVID-19, as reported by several research groups, the enhanced formation of NET is associated with fatal outcomes [47, 123–125]. Therefore, LaSalle et al. hypothesized that in the early stages, IgA induced NET release is beneficial for preventing SARS-CoV-2 entry into the mucosal region, while NET release may be harmful to circulation and promote tissue damage in the later stage [48]. This provides a breakthrough for the development and application of COVID-19 therapy based on IgA.

### SIgA and lung cancer

In many human malignancies, such as lung cancer, gastrointestinal cancer, and hematopoietic cell cancers, studies have confirmed that the expression level of SIgA in patients with disease, is different from that in healthy people [126, 127]. Therefore, it has been further proposed by early relevant studies that the secreted IgA antibody is specific for tumor cell clearance, and the relevant immune response can be used for the early detection of cancer [128]. The IgA antibody isolated or screened from tissues can directly act on tumor cells in vitro or in vivo, so it may be used for tumor treatment [129]. IgA deficiency was significantly associated with cancer risk and cancer mortality [50, 51]. It has been found that, compared with patients without neoplastic bronchopulmonary disease, 30% of patients with lung cancer, especially those with squamous cell carcinoma, have increased levels of SIgA synthesis in normal bronchial tissues. The determination of SIgA in bronchial mucosa can be regarded as a valuable early diagnostic aid for suspected lung tumors [130, 131]. The decreased concentration of SIgA in the airway of lung cancer patients may have adverse effects on airway resistance to bacterial colonization [132].

However, the relationship between SIgA and lung cancer is complex and subject to confusion and reverse causation. On one hand, low SIgA levels were significantly associated with cancer risk and cancer mortality [50, 51], possibly because of reduced immune surveillance of mucosal sites and glands discharging into the mucosa. On the other hand, a high level of SIgA is considered to be an indicator of an existing underlying disease. At some point, immune cells in lung cancer patients interact with tumor cells to cause an immune response that promotes SIgA production. Moreover, since the occurrence of lung cancer is closely linked to environmental factors [50], other lung diseases caused by environmental factors may also detect an increase in SIgA. Therefore, the measurement of SIgA is unlikely to be clinically significant in the diagnosis of lung malignancy. Atis et al. found that the SIgA level of bronchial lavage fluid in chronic bronchitis, bronchiectasis, and lung cancer groups was no significant difference between the groups. They believed that SIgA was not useful in distinguishing the respiratory epithelial injury or inflammatory reaction of patients with different pulmonary diseases [133]. At present, SIgA has not been used as a potential breakthrough in the diagnosis and treatment of lung cancer. However, perhaps, there will be discoveries in the future, which need further research.

## Conclusion

To sum up, SIgA is involved in the pathogenesis and progression of various lung diseases and has complex and effective immune functions. With the advancement of this field, SIgA can serve as a potential target for the treatment of lung diseases. However, there have been few previous studies using SIgA as a therapeutic antibody, and there is a lack of evidence at both preclinical and clinical levels on whether SIgA-related antibodies can provide clinical benefits for patients with airway diseases. Preliminary studies have shown that intervention on the level of SIgA in lung diseases can delay the progress of the disease to a certain extent. SIgA can be used as a promising biomarker. Through further exploration of its functions, receptor complexes, induced effects and mechanisms, related drugs, and their interaction, we believe that the mechanisms of SIgA in lung diseases will be gradually clarified, which can provide new ideas and targets for the prevention, treatment and diagnosis of various lung diseases. In addition, with the further development of recombinant antibody production, complex and polymeric SIgA molecules may be produced on a large scale and with high quality, thus making the widespread application of therapeutic SIgA formulations possible.

## Data Availability

Not applicable: the data sets generated and/or analysed during the current study are available in the PubMed.

## Abbreviations

SIgA: Secretory immunoglobulin A
IgA: Immunoglobulin A
dIgA: Dimer IgA
SC: Secretory component
fSC: Free secretory component
pIgR: Polymeric immunoglobulin receptor
M cells: Micro pleated epithelial cells
COPD: Chronic obstructive pulmonary disease
SIgAD: Selective immunoglobulin A deficiency
BALF: Bronchoalveolar lavage fluid
IPF: Idiopathic pulmonary fibrosis
IL: Interleukin
MCP: Monocyte chemoattractant protein
VEGF: Vascular endothelial growth factor
TGF: Transforming growth factor
EMT: Epithelial mesenchymal transition
TfR: Transferrin receptor
SARS-CoV-2: Severe acute respiratory syndrome coronavirus 2
